# Primary Central Nervous System Lymphoma as a Masquerade of Uveitis: A Case Report of Ocular Involvement

**DOI:** 10.7759/cureus.104790

**Published:** 2026-03-06

**Authors:** Sue-Zian Goh, Kwang-Sheng Ng, Wan-Hazabbah Wan Hitam, Sruban Suparmaniam, Mohammad Hudzaifah Nordin

**Affiliations:** 1 Department of Ophthalmology and Visual Science, School of Medical Sciences, Universiti Sains Malaysia, Kubang Kerian, MYS; 2 Department of Ophthalmology, Hospital Sultanah Bahiyah, Alor Setar, MYS; 3 Department of Ophthalmology, Hospital Teluk Intan, Teluk Intan, MYS; 4 Faculty of Medicine, Universiti Sultan Zainal Abidin, Kuala Terengganu, MYS

**Keywords:** deangelis protocol, leopard-skin retinopathy, masquerade syndrome, primary central nervous system lymphoma (pcnsl), vitreoretinal lymphoma

## Abstract

Primary central nervous system lymphoma (PCNSL) often presents with ocular involvement that mimics benign inflammatory conditions, frequently delaying diagnosis. We report a case of a 41-year-old healthy woman presenting with diminished vision and floaters. Examination revealed panuveitis with characteristic hypopigmented retinal lesions exhibiting a 'leopard-skin' pattern. Despite the absence of systemic symptoms and a vitreous sample of low cellularity, diagnostic pars plana vitrectomy confirmed large B-cell lymphoma via immunophenotyping (CD20, CD79a and PAX5). Subsequent magnetic resonance imaging (MRI) demonstrated a parieto-occipital brain mass.

The patient received high-dose methotrexate-based chemotherapy according to the DeAngelis protocol, combined with rituximab, selected to optimise disease control while minimising the neurotoxicity associated with whole-brain radiotherapy. She achieved visual recovery to 6/9 and regression of the central nervous system (CNS) lesion. This case underscores the importance of recognising pathognomonic retinal signs and employing molecular immunophenotyping to enable timely, life-saving, neuro-sparing therapy.

## Introduction

Primary central nervous system lymphoma (PCNSL) is a rare, aggressive extranodal non-Hodgkin lymphoma affecting the brain, leptomeninges, eyes or spinal cord. Although it accounts for only about 4% of brain tumours, its incidence has increased steadily, particularly among individuals aged over 60 years [1]. A distinct subtype, primary vitreoretinal lymphoma (PVRL), involves the malignant lymphoid infiltration of the vitreous and subretinal spaces.

PVRL is often described as a masquerade syndrome, as blurred vision, floaters and vitritis can mimic benign posterior uveitis. This clinical overlap, along with inappropriate corticosteroid use, which may provide only temporary improvement, can mask the disease and result in diagnostic delays. Early recognition is crucial, as 65%-90% of the patients with PVRL eventually develop central nervous system (CNS) involvement [2].

Diagnosis primarily relies on vitreous cytology, but interpretation requires considerable expertise, as specimens often contain few neoplastic cells that may be outnumbered by reactive elements. Fragile tumour cells and the inaccessibility of subretinal lesions further complicate analysis, contributing to frequent false-negative results. Multiple specimens are often necessary, and the diagnosis of PVRL is frequently delayed [3].

We report a case of PCNSL in an immunocompetent woman presenting with panuveitis and 'leopard-skin' retinopathy. Diagnosis was confirmed through molecular immunophenotyping, and the patient responded favourably to a neuro-sparing chemotherapy regimen.

## Case presentation

A 41-year-old previously healthy woman presented with a four-day history of reduced vision and floaters in the right eye. This was her first episode of ocular symptoms, with no history of ocular trauma. Systemic review was unremarkable, with no neurological, respiratory or constitutional symptoms.

**Figure 1:**
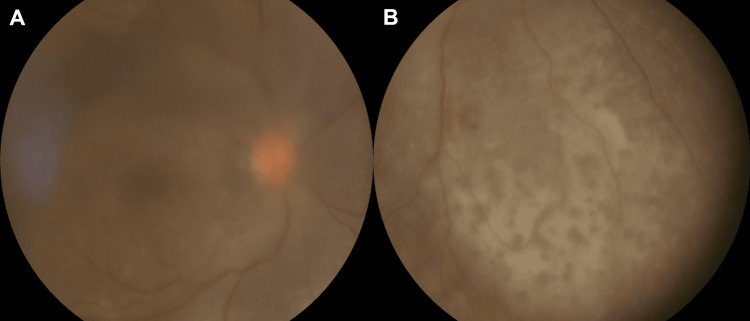
(A) Wide-field fundus image of the right eye showing dense vitritis (3+ haze) obscuring fine retinal details. (B) Multiple flat, patchy hypopigmented subretinal infiltrates are visible along the inferonasal arcade. These lesions exhibit the characteristic 'leopard-skin' pattern, which is considered pathognomonic for PVRL. PVRL: primary vitreoretinal lymphoma

Best-corrected visual acuity was 6/24 in the right eye and 6/6 in the left eye, with an intraocular pressure of 14 mmHg. The slit-lamp examination of the right eye showed granulomatous keratic precipitates with 1+ anterior chamber cells and flare. Fundus examination revealed dense vitritis (3+) obscuring retinal details, with a flat, patchy hypopigmented lesion exhibiting a characteristic 'leopard-skin' appearance along the inferonasal arcade (Figure 1). The left eye was normal.

Laboratory investigations (Table 1) demonstrated a normal haematological profile, with an elevated C-reactive protein of 39 mg/L. Infectious screening was negative. The Mantoux test was negative as well. Fundus fluorescein angiography showed masking from vitritis without leakage.

**Table 1:** Laboratory investigations demonstrated a normal haematological profile and negative infective screening. C-reactive protein was raised. Reference ranges for full blood count and C-reactive protein obtained from the local pathology handbook [4]. CMV, cytomegalovirus; HSV, herpes simplex virus; RPR, rapid plasma reagin; HBsAg, hepatitis B surface antigen; HCV, hepatitis C virus; HIV Ag/Ab, human immunodeficiency virus antigen/antibody; NA, not applicable

Parameter	Result	Normal Range	Unit
Haemoglobin	13.6	12.0-15.0 (female)	g/dL
Total white blood cell	6.5	4.0-10.0	10^3^/µL
Platelet count	235	150-410	10^3^/µL
C-reactive protein	39	<5	mg/L
Toxoplasma IgM	Non-reactive	NA	-
Toxoplasma IgG	Non-reactive	NA	-
CMV IgM	Non-reactive	NA	-
CMV IgG	Non-reactive	NA	-
HSV-1 IgG	Non-reactive	NA	-
HSV-2 IgG	Non-reactive	NA	-
RPR	Non-reactive	NA	-
HBsAg	Non-reactive	NA	-
Anti-HCV	Non-reactive	NA	-
HIV Ag/Ab	Non-reactive	NA	-

**Figure 2:**
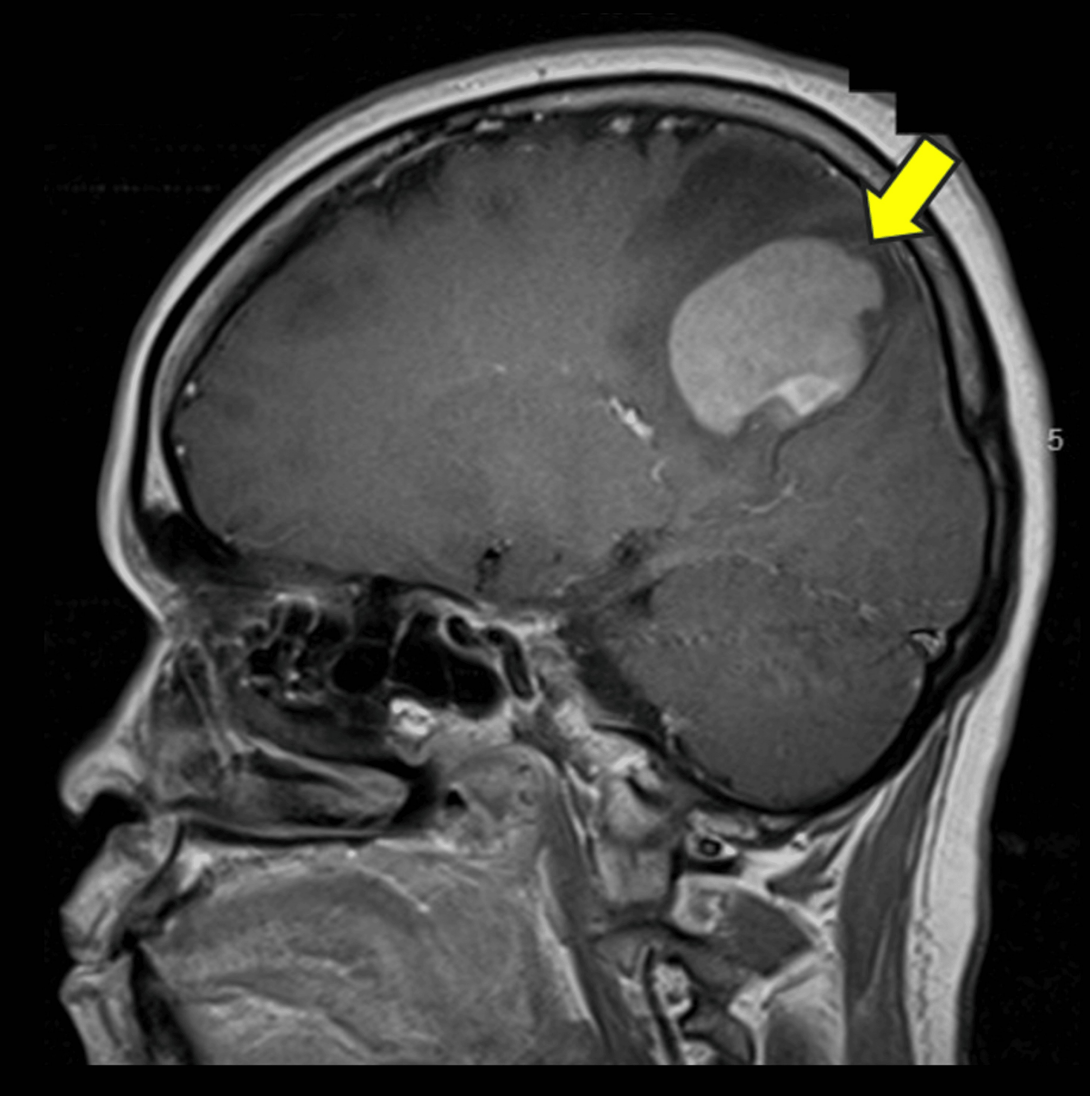
Sagittal T1-weighted contrast-enhanced MRI at presentation, showing a well-defined hyperintense intra-axial mass in the right parieto-occipital lobe (yellow arrow) at the parasagittal region. MRI: magnetic resonance imaging

A diagnostic pars plana vitrectomy was performed. Despite low cellularity, vitreous cytology revealed atypical B-lymphoid cells. Immunophenotyping confirmed CD20, CD79a and PAX5 positivity, consistent with large B-cell intraocular lymphoma. Magnetic resonance imaging (MRI) of the brain identified a parieto-occipital intra-axial mass (Figure 2).

A diagnosis of primary central nervous system lymphoma with ocular involvement was made. The patient was treated with systemic chemotherapy according to the DeAngelis protocol and rituximab. Visual acuity improved to 6/9 with the resolution of panuveitis and the regression of retinal lesions. Follow-up neuroimaging showed tumour reduction. At 18 months, the patient remains clinically stable with ongoing surveillance.

## Discussion

This case illustrates the diagnostic challenge of PCNSL, a masquerade syndrome in which a life-threatening malignancy mimics benign inflammatory ocular disease. Delayed diagnosis is common, typically occurring 8-21 months after symptom onset [3]. In this patient, granulomatous keratic precipitates and vitritis resembled common uveitis, but the recognition of characteristic fundus features and early molecular diagnostics were critical in securing the correct diagnosis.

PVRL typically presents as creamy yellow-white subretinal or sub-retinal pigment epithelium (RPE) infiltrates of varying size, with pigment islands forming characteristic 'leopard-spot' patterns. Irregular solid RPE detachments with deposits are considered pathognomonic, and the recognition of this pattern is particularly important in patients without neurological symptoms, such as our patient [5].

Vitreous biopsy remains the diagnostic cornerstone but is limited by low cellularity and fragile lymphoma cells, yielding sensitivities of 45%-81% [6]. The sensitivity of cytological diagnosis can be improved by complementing it with immunocytochemical staining, which uses antibodies to detect cell type-specific markers, such as B-cell markers CD20, CD79a and PAX5 [7,8]. In this case, the immunophenotyping of a sparse vitreous sample confirmed the expression of these markers, consistent with B-cell lineage.

MRI revealed a parieto-occipital intra-axial mass, emphasising the high likelihood of CNS involvement in ocular presentations, which occurs in about 65%-90% of cases [2]. This supports the inclusion of contrast-enhanced MRI in the workup of atypical uveitis.

The patient received high-dose methotrexate-based chemotherapy according to the DeAngelis protocol, combined with rituximab, achieving visual recovery to 6/9 and the regression of ocular and CNS lesions. This outcome demonstrates the efficacy of the DeAngelis protocol, which was developed to optimise the effectiveness of chemotherapy [9].

## Conclusions

The recognition of characteristic ocular features, such as 'leopard-skin' subretinal lesions, is critical in patients with atypical uveitis. The early diagnosis of primary vitreoretinal lymphoma, supported by vitreous biopsy and molecular immunophenotyping, allows timely intervention even without neurological symptoms. Contrast-enhanced MRI is essential to detect CNS involvement. High-dose methotrexate-based chemotherapy, combined with rituximab, can achieve both visual and systemic disease control while minimising neurotoxicity, emphasising the importance of prompt, targeted management in this aggressive malignancy. Nevertheless, there are inherent limitations, as this is a single case report.
